# Evaluation of rehabilitation effect of five-step exercises on patients with radiculopathy of cervical vertebra

**DOI:** 10.1097/MD.0000000000020846

**Published:** 2020-06-26

**Authors:** Xia Xu, Yajie Wang, Chen Yang, Xinyu Song, Zhilong Chen, Lingge Yang, Yaxin Li

**Affiliations:** a Gansu Provincial Hospital of Traditional Chinese Medicine; b Gansu University of Chinese Medicine; c Lanzhou University, Lanzhou, Gansu, China.

**Keywords:** cervical spondylosis, cervical spondylotic radiculopathy, protocol

## Abstract

**Background:**

Among all types of cervical spondylitis, cervical spondylitis radiculopathy (CSR) has the highest incidence. The incidence of CSR increases year by year and is generally younger, which has seriously threatened people's quality of life and affected their work and life. This study proposes to improve the recovery rate of patients with CSR, delay the recurrence, improve the symptoms of patients, and improve the quality of life of patients through the rehabilitation and exercise of five-step cervical vertebra exercises.

**Methods:**

For 90 patients with CSR that met the inclusion criteria, SPSS 23.0 software random number generator was used to randomly divide the patients into an experimental group and control group, with 45 cases in each group. The control group took basic nursing measures, and the experimental group took five steps of cervical vertebra rehabilitation exercises on the basis of elementary nursing measures. The rehabilitation effect of five-step exercises on CSR patients was evaluated by Neck Disability Index (NDI), Visual Analogue Scale (VAS), and Cervical range of motion measured (CROM) before and after intervention.

**Results:**

The results of this trial will be published on the website of China Clinical Trial Registration Center (http://www.chictr.org.cn/searchproj.aspx) and in peer-reviewed journals or academic conferences.

**Conclusions:**

This study will examine the feasibility and preliminary effects of five-step exercises for the treatment of patients with CSR.

**Trial registration:**

This protocol was registered in Clinical Trials platform with the number ChiCTR1900027299.

## Abstract 2

Supplemental Digital Content is available in the text

## 1 Introduction

Cervical spondylotic radiculopathy (CSR) is a neurological disease characterized by cervical spinal nerves, nerve roots, or bilateral dysfunction.^[1]^ Compression of nerve roots can be due to disc herniation. Pain is typically manifested by unilateral arm pain around the scapula and upper back pain, and sometimes by arm weakness.^[2,3]^ The incidence of CSR is about 1.79/1000 persons/year, and the patients are mainly between 30 and 50 years old.^[4,5]^ Due to the changes in people's lifestyle, the prevalence of cervical spondylitis increases year by year and shows a trend of being younger.^[6]^

Currently, surgical treatment and non-surgical treatment (including drugs, traction, physical therapy, functional exercise, etc) are utilized to treat CSR. The relevant guidelines^[7]^ point out that non-surgical treatment is normally adopted for CSR patients, but surgical treatment is recommended when conservative treatment is ineffective or although effective, it still occurs repeatedly and seriously affects the patients’ quality of life. When the neck is in a state of tension, can make the muscle tissue swelling, stiffness, and decreased muscle strength after extensor group is particularly significant, at the same time because of the long-term abnormal force on both ends of the muscle attachment points of tension, the cervical curvature change, lead to cervical instability, disc herniation, joint capsule edema, and so on, eventually CRS related symptoms.^[8]^ A large number of studies have shown that^[9–12]^: through the training of neck muscles in different directions and angles, sports therapy can enhance the strength of neck muscles and thus relieve neck pain, effectively improve neck function and relieve the clinical symptoms of cervical spondylitis.

**Figure 1:**
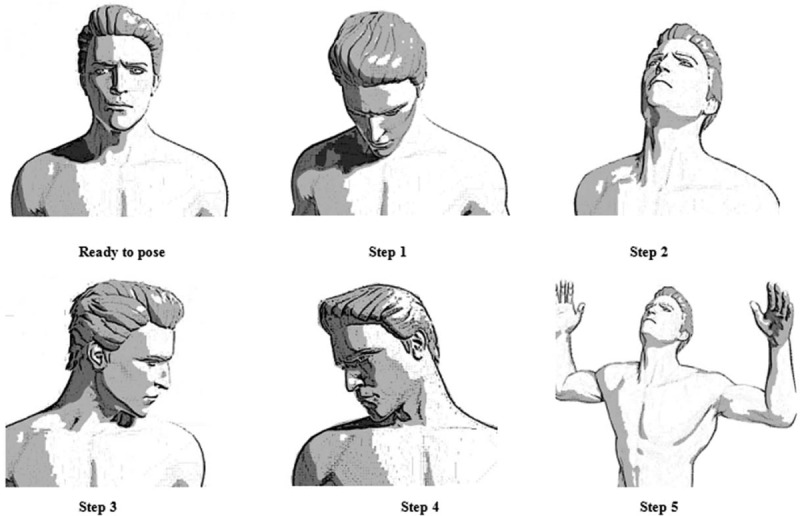
Schematic diagram of cervical five-step exercises.

Exercise as an alternative treatment for cervical spondylosis is popular in China. Cervical spine five-step exercises (Fig. 1) are selections of 5 simple and effective movements from various sports therapies, including looking down at the feet, looking up at the sky, looking at the shoulder peak to the left, looking at the shoulder peak to the right, and expanding the chest, looking up and bending the elbow. Compared with other sports therapies, it has the simple operation and more relaxing movements. The purpose of this study was tantamount to investigate the initial efficacy of cervical spine five-step exercises in the treatment of CSR.

## 2 Methods

### 2.1 Study design

The present study is a single-blind, randomized controlled clinical study that applies the ethical principles of China's Ministry of Health's “Ethical Review Measures for Biomedical Research Involving Human Beings (Trial 2007),” “Regulations on Clinical Trials of Medical Devices (2004),” WMA's “Helsinki Declaration,” and CIOMS's “International Ethical Guidelines for Biomedical Research on Human Beings.” The project was evaluated and approved by the Research Ethics Committee of Gansu Provincial Hospital of Traditional Chinese Medicine (Ethical batch No. FJ/ 01-irb /C/ 018-v3.0). Treatment will be held in Gansu Provincial Hospital of Traditional Chinese Medicine from June 1, 2020 to June 1, 2021. The schedule of pre-intervention, intervention and evaluation procedures will be provided in Supplemental Material 1. A flow chart representing the stages of this study is shown in Supplemental Material 2.

### 2.2 Participants

The following eligibility criteria are applied to all patients for recruitment into the study. Patients will be recruited through the inpatient orthopedic department of Gansu Provincial Hospital of Traditional Chinese Medicine. After oral explanation and reading of the procedures used in the study, patients agreeing to participate will sign an informed consent form.

#### 2.2.1 Eligibility

- It conforms to the diagnostic criteria of CSR issued by Chinese rehabilitation medical association^[13]^ (intervertebral foramen compression test, brachial plexus traction test, pain or numbness in the upper extremity are all considered to be diagnosable);
- Between the ages of 18 and 65;
- Conservative treatment of CSR;
- Not participating in other clinical trials during this period;
- Patients who voluntarily participated in this study and signed the informed consent.

#### 2.2.2 Ineligible

- Pregnant or nursing women;
- Serious cardiovascular, liver, kidney, hematopoietic system and malignant tumors, severe trauma, sequelae of cerebral hemorrhage, and other serious primary;
- Suffering from spinal instability lesions (spinal tuberculosis, fracture, bone tumor, and rheumatoid arthritis), cervical congenital deformities, acute tissue injury;
- Damaged integrity of the neck skin;
- Mental patients or do not cooperate with patients;
- Non-radiculopathic cervical spondylitis patients;
- Patients with CSR receiving surgical treatment.

### 2.3 Interventions

#### 2.3.1 Control group

- Pain care: nonsteroidal anti-inflammatory drugs or opiate analgesics can be given to patients with pain.
- Diet nursing: patients with CSR generally take general food as the main food, and eat more high-fiber and digestible food. When patients need bed rest, avoid greasy food to prevent abdominal distension and constipation, and keep drinking 1500 to 2000 mL water every day.
- Life care: when sleeping pillow height is 10 to 15 cm, the neck cannot hang up, to keep the head back slightly. Longbow, long-term fixed position work pays attention to the mix, appropriate to the neck. Pay attention to the neck to keep warm, avoid long time blow air conditioning, electric fan. Reduce neck strenuous exercises and prevents injuries.
- Psychological nursing: due to the protracted and repetitive course of CSR, it is very easy for patients to have bad emotions such as impatience, anxiety, and pessimism in their daily life. Nursing staff should use psychological nursing intervention, through a variety of communication skills to communicate with patients, understand the patient's psychology, observe the changes in mood, timely adjust the patient's sour mood.
- Health education: carry out health education for patients, so that they can understand the pathogenesis, prevention and treatment of cervical CSR and prevent recurrence.

#### 2.3.2 Observation group

On the basis of pain nursing, diet nursing, life nursing, psychological nursing, and health education, rehabilitation training of five steps of cervical vertebra was carried out twice a day during hospitalization. The following five steps were performed for 1 time, and the second time was performed after resting for 1 min, each time lasting 10 to 15 min.

Method of five-step cervical spine operation: patients should take a standing position, stand with their feet shoulder-width apart, look forward, breathe well, and then do the following actions:

- Slowly lower your head, look at your toes, keep your lower jaw as close to the sternum as possible, hold it for 5 to 10 s, and then return to the ready position;
- Slowly raise your head and lean back to the maximum, 5 to 10 s, then return to the ready position;
- Turn your head slowly to the left, and look at your left shoulder to the maximum, 5 to 10 s, then return to the ready position;
- Turn your head slowly to the right, and look at your right shoulder to the maximum, 5 to 10 s, then return to the ready position;
- Lift up your arms to shoulder level and bend your elbows with your chest extended, pull your neck back as far as possible during chest expansion, 5 to 10 s, and then return to the preparation position.

### 2.4 Sample size calculation

NDI is the independent primary outcome measure and sample size estimate was carried out for outcome measures. An minimal clinically important difference (MCID) for NDI of 7 has been determined for cervical radiculopathy. Using a reported NDI standard deviation (SD) of 9.2^[14]^ for this patient group, with a two-sided 5% significance level and a power of 80%, a sample size of 39 participants per group is required. Considering a 15% dropout rate, a total of 90 cases were recruited, 45 in each group.

### 2.5 Randomization and allocation concealment

Ninety patients will be split into experimental and control groups with a ratio of 1:1. A statistical expert not involved in the design of the study used SPSS 23.0 software to generate a set of random sequences, to be sealed in opaque envelopes prepared in advance. Only 4 nurses were allowed to open the envelopes to obtain each grouping code.

### 2.6 Single-blinding

Nurses were not provided details of treatment plans or grouping information within the envelope in advance. No participant or relevant medical personnel was authorized to interfere with individual treatment selections. Evaluators are blinded to the treatment each patient has received, their function being to assist each patient in completion of the various questionnaires and scoring the respective scales. It is intended that the independent biostatisticians are also blinded when conducting the statistical analysis.

### 2.7 Rejection, dropout, and termination criteria

- Those who are found not in conformity with the inclusion criteria by further examination after inclusion;
- During the observation period accidental disease need to receive other treatment;
- Data is not completely unable to determine the curative effect;
- Do not cooperate, quit;
- Incomplete treatment;
- Due to adverse reactions or other reasons to stop the original treatment plan;
- Follow up those who fall off.

### 2.8 Outcomes

#### 2.8.1 Primary outcome

##### 2.8.1.1 NDI

As a measure of neck-specific functional disability, a translated version of the original 10-item Neck Disability Index (NDI) will be used. The NDI covers 10 dimensions of neck-specific disability, namely pain intensity, personal care, lifting, reading, headache, concentration, work, driving, sleeping, and recreation.^[15]^ Each item assesses one dimension and is measured on a 6-point scale from 0 (no disability) to 5 (highest disability). The overall score (out of 100) is calculated by adding the score for each item and multiplying by 2.^[16]^ A higher score indicates greater pain and disability.^[17]^ Before treatment, after each week of treatment, and after treatment, the evaluators assessed and recorded.

#### 2.8.2 Secondary outcomes

##### 2.8.2.1 VAS^[18]^

The VAS (0–10) was used to evaluate the intensity of pain. The self-report questionnaire was carried out the treatment process according to the patients’ subjective feeling. Before treatment, after each week of treatment, and after treatment, patients were rated on a visual simulation scale to assess their pain level (from no pain to very severe), which was then registered with the evaluator.

##### 2.8.2.2 CROM^[19]^

Cervical range of motion measured with a CROM device (Performance Attainment Associates, USA). Flexion, extension, side flexion, and rotation, along with symptom response, are being measured. Before treatment, after each week of treatment, and after treatment, the evaluators assessed and recorded.

### 2.9 Adverse events and treatment plan

Against five steps exercises rehabilitation training in patients with cervical spine during show a variety of adverse reactions to register a detailed classification, specific content have adverse symptoms, occurrence time, duration, degree, and the treatment measures, etc, and record in the cases of adverse reaction when doctors treatment measures, according to the chief physician to decide whether to continue to participate in the test.

### 2.10 Data management

After data collection is completed, all data will be recorded independently by two trained research assistants using paper case report forms (CRFs). Independent investigators employed by the hospital will regularly monitor and review the experimental data. The final data will be uploaded to the ResMan clinical trials public management platform through the China clinical trials registry.

### 2.11 Statistical analysis

Statistical analysis will be performed using SPSS23.0 software. The difference between the two groups will be calculated and compared using the *t* test if the Shapiro–Wilk test showed that the data are normally distributed. Otherwise the Mann–Whitney *U* test will be used. A Pearson χ^2^ or Fisher's exact test will be used to calculate the differences in the count data.

### 2.12 Ethics and dissemination

This study has passed the ethical review of the experimental unit (approval document no. FJ/01-irb /C/ 018-v3.0). Written informed consent will be obtained from all of the participants or their authorized agents.

## 3 Results

The results of this trial will be published on the website of the China Clinical Trial Registration Center and in peer-reviewed journals or academic conferences.

## 4 Discussion

There are many types of conservative treatments, including traction, exercise therapy, physical therapy, etc. Among them, exercise therapy is cheap, easy to operate, easy to be accepted by patients, and has been proved to be effective in alleviating the symptoms of CSR patients,^[20,21]^ compared with surgical treatments with large trauma, high cost, and postoperative complication risk. Cervical vertebra operation is a kind of kinesiotherapy, which has a fair prospect in relieving the symptoms of patients. The orthopedic of traditional Chinese medicine theory holds that both the static system (ligaments, joint capsules, etc) and the dynamic system (muscles, intervertebral discs, small joints, etc) are critical in maintaining normal position and function of the cervical spine. The imbalance of both static and dynamic forces can result in a loss of posterior column stability, ultimately leading to rapid degeneration of the cervical intervertebral discs and causing a series of syndromes distributing along the spinal nerve roots (such as pain, numbness of the neck, shoulder, and arm, etc).^[22,23]^ Mechanism of action of cervical vertebra^[24]^: through cervical vertebra movement, muscle spasm can be relieved, muscle strength balance can be restored, blood supply can be improved, ligament morphology and structure can be restored and inherent regulation function can be restored, finally achieving the balance between static system and dynamic system. Other scholars have found that^[25]^ conditioned pain can be activated through neck exercises to regulate the downward inhibitory pathway, thereby reducing pain.

This study has a few limitations. First of all, there are differences in gender, age, occupation and other aspects of the patients, so it may be necessary in order to conduct subgroup analysis in the future to judge the effectiveness of five-step cervical spine exercises in different subgroups. Secondly, as indicators such as VAS and NDI are subjective, patients’ education level, cognitive ability and degree of cooperation may affect the results, making the results biased. Finally, cervical spondylitis can be divided into five types. Since CSR accounts for the highest proportion, this study evaluated the efficacy of five-step exercises for patients with CSR. The efficacy of five-step exercises on other types of cervical spondylitis and neck fatigue in young and middle-aged people remains to be confirmed by future studies.

## Author contributions

**Conceptualization**: Xia Xu, Yajie Wang and Chen Yang

**Data curation**: Lingge Yang and Xinyu Song

**Formal analysis**: Xia Xu and Zhilong Chen

**Funding acquisition**: Xia Xu

**Investigation**: Yajie Wang, Chen Yang and Yaxin Li

**Project administration**: Xia Xu

**Supervision**: Xia Xu, Yajie Wang and Chen Yang

**Writing – original draft**: Yajie Wang and Chen Yang

## Supplementary Material

Supplemental Digital Content
