# Supplementary material for: Evaluation of rehabilitation effect of five-step exercises on patients with radiculopathy of cervical vertebra
Source: Medicine (Baltimore). 2020 Jun 26;99(26):e20846. doi: 10.1097/MD.0000000000020846 (PMC7328935; doi:10.1097/MD.0000000000020846)
Supplement: Supplemental Digital Content [file medi-99-e20846-s001.pdf]

Material 1 The schedule of trial enrolment, interventions and assessments

	Enrolment	Intervention period			Follow-up after discharge period			06/2021 to 09/2021
		0	7 day after treatment	14 day after treatment	30 days after discharge	60 days after discharge	90 days after discharge	
**Enrolment**								
Informed consent	•							
Assessment of eligibility	•							
Randomisation	•							
**Interventions**								
Control group			•	•	•	•	•	
Observation group			•	•	•	•	•	
**Assessments**								
NDI		•	•	•	•	•	•	
VAS		•	•	•	•	•	•	
Cervical range of motion measured		•	•	•	•	•	•	
**Statistical analysis**								•

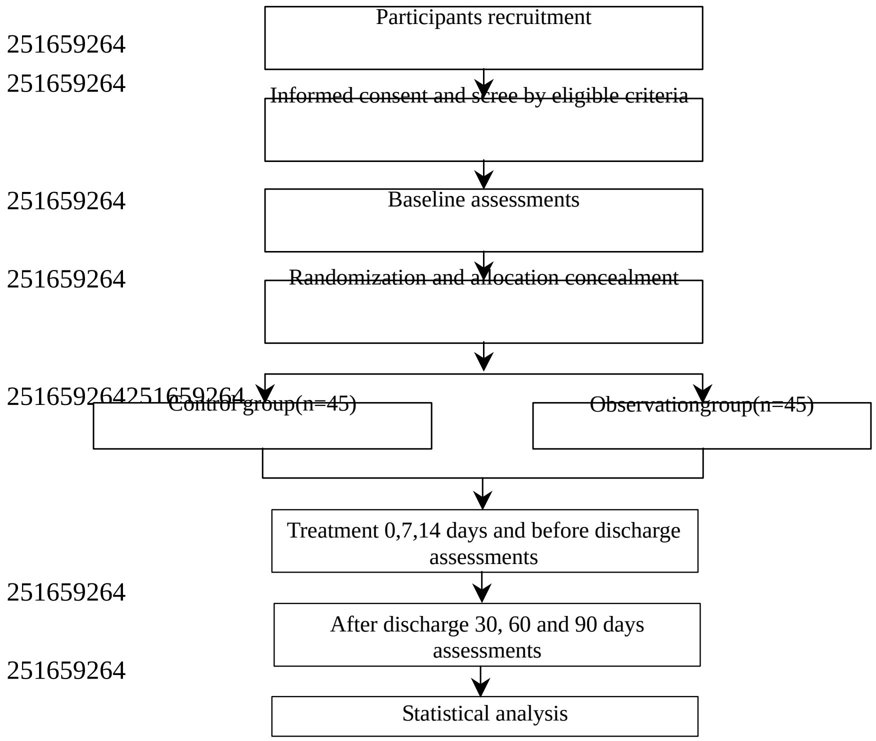

Material 2 The study flow diagram, including participants' recruitment, eligibility, screening, randomisation, allocation concealment and outcome assessments.
